# HEAD COMPUTED TOMOGRAPHY EXAMINATION AS A FACTOR OF RADIATION EXPOSURE IN CHILDREN TREATED FOR HYDROCEPHALUS

**DOI:** 10.13075/ijomeh.1896.02572

**Published:** 2025

**Authors:** Michał Biegała, Krystian Skoczylas, Katarzyna Matera, Piotr Grzelak, Maria Anna Staniszewska

**Affiliations:** 1 Medical University of Lodz, Faculty of Medicine, Department of Medical Imaging Technology, Łódź, Poland; 2 Polish Mother's Memorial Hospital Research Institute, Department of Diagnostic Imaging, Łódź, Poland

**Keywords:** radiation protection, effective dose, CT examination, hydrocephalus, probability of induction of leukemia, dose in CT

## Abstract

**Objectives:**

Computed tomography (CT) in children with hydrocephalus is a procedure often performed from the first days of the child's life. It is important in diagnosing and monitoring treatment progress.

**Material and Methods:**

Based on a retrospective analysis of CT scans, the level of exposure to ionizing radiation in children with hydrocephalus subjected to this study was calculated. The probability of induction and death from leukemia or other cancers as a result of CT scans was also calculated.

**Results:**

The highest exposure is observed in children <1 year of age: M±SD 4.2±0.9 mSv/year. In the following years, this exposure decreases, reaching the level of 0.7±0.1 mSv/year at the age ≥11 years. This is correlated with the probability of induction of leukemia and other cancers, which is highest in the first year of life. In subsequent years, the probability decreases. The probability of dying from these cancers remains at a similar level all the time. By the age of 17 years, a patient with hydrocephalus diagnosed in infancy may receive a total effective dose of almost 21 mSv.

**Conclusions:**

After analyzing exposure over the years, a significant reduction in the number of CT examinations performed and a reduction in the radiation dose received by children was found through the introduction of pediatric CT examination protocols.

## Highlights

- The number of head computed tomography (CT) performed in children has been decreasing in recent years.
- Pediatric protocols significantly minimize children's exposure during head CT.
- In diagnosing the highest doses are given to children <1 year of age.

## INTRODUCTION

Imaging tests are an integral part of modern diagnostics. Particular attention is required for diseases during the treatment of which imaging tests must be repeated many times to monitor the treatment process. Hydrocephalus is one of such diseases.

Hydrocephalus is a neurological disease that causes damage to the balance between nervous tissue, cerebrospinal fluid and vascular bed, which leads to an increase in intracranial pressure and excessive accumulation of cerebrospinal fluid in the ventricular system of the brain.

The balance between the production and absorption of cerebrospinal fluid is extremely important, because it performs very important functions in the body. It acts as a shield for the cerebral cortex, providing mechanical and immunological protection inside the skull, and also plays an important role in cerebral circulation.

The obvious symptoms of the disease are a sharp increase in head circumference, bulging and widening of the fontanel. Other symptoms include: vomiting, drowsiness, irritability, convulsions, as well as the “sunsetting” symptom, in which the eyeballs are turned down when looking up. Hydrocephalus has many causes:

– it may be congenital hydrocephalus, i.e., existing from the moment of birth, which is related to genes regulating the growth and development of the brain and most often involves narrowing of the aqueduct;
– can be acquired and may occur at any stage of a person's life as a result of an accident, surgery, cerebral bleeding, tumor, meningitis and/or other causes – mainly as a result of pathological processes that affect ventricular outflow, the function of the subarachnoid space or susceptibility of cerebral veins.

During the treatment of hydrocephalus, X-ray computed tomography (CT) is most often performed. Although it is related to exposure to X-rays, this test is repeated many times during the treatment process to control the effectiveness of this process [1–7]. In CT examinations, the radiation dose is received not only by the head area, but also by other organs that are not imaged during head CT [8]. A large number of CT repeats in childhood can significantly influence the probability of cancer induction later in life and lead to death as a result of its induction [9].

The aim of this analysis is to assess the exposure to ionizing radiation in children who were treated for hydrocephalus and repeatedly examined using a CT scanner, and to estimate the risk of induction and death due to leukemia or other types of cancer.

## MATERIAL AND METHODS

The analyzed group of patients included children and adolescents <18 years of age who were treated for hydrocephalus at pediatric reference center in January 2021 – August 2022 and during the treatment process were subjected to CT of the head. The tests were performed using a Canon GENESIS ONE 640 TSX-305 A/5 CT scanner (Canon Medical Systems, Ōtawara, Japan; manufactured in 2019). All head CT examinations in children at the pediatric reference center were performed in accordance with applicable medical procedures for pediatric patients [10].

Patients and their parents/carers were informed about the procedure for performing a head CT examination. Head CT examination is usually performed without anesthesia, but the patient's cooperation is required during the examination. The patient must remain in the position determined by the electroradiologist conducting the examination, do not say anything, do not move. This is crucial because only in this way the electroradiologist is able to achieve the required diagnostic image quality; otherwise, artifacts will be created that make it impossible to detect pathology, and the test will have to be repeated. To avoid such situations, in the case of small children and uncooperative children, it is sometimes necessary to use general anesthesia, thanks to which the small patient remains still and the diagnostic process proceeds properly.

Patients received protective shields on their thyroid, chest, abdomen and pelvis for the duration of the study. Care was also taken to protect parents who accompanied young patients during head CT examinations, and women of childbearing age were informed that they could not accompany their children during the examination if they were or were likely to be pregnant.

All radiological examinations described in this work were performed in accordance with the generally accepted reference level regarding radiation doses. The Table 1 shows the range of pediatric head CT dosimetry reference values for the Canon scanner.

**Table 1.** Reference dosimetric values for head computed tomography protocols performed with a Canon scanner for age groups

Age group	Reference dosimetric value	
	DLP [mGy × cm]	CTDI_vol_ [mGy]
0–2 years	278.7	19.8
3–5 years	495.4	40.5
6–12 years	568.7	40.5
13–16 years	778.4	55.4
≥17 years	≥978.0	59.1

CTDI_vol_ – computed tomography dose index; DLP – dose-length product.

The head CT examination protocol was performed in accordance with the current recommendations for pediatric head CT examinations [10].

In the analysis, patients were divided into 4 age groups: <1 year, 1–3 years, 4–10 years and ≥11 years. This division reflected the frequency of tests ordered by neurologists in the treatment process and monitoring its effects. Dose length product (DLP) (mGy × cm), CT dose index CTDI_vol_ (mGy), peak kilovoltage (kVp), milliampere-seconds (mAs)/tube revolution were recorded for all patients; all CTDI_vol_ values reported in dose reports were based on a 16 cm diameter phantom. The exposure parameters of the head CT scans were as follows:

– age 0–2 years – 135 mAs and 100 kV,
– age 3–5 years – 240 mAs and 100 kV,
– age ≥6 years – 337 mAs and 100 kV or 225 mAs and 120 kV,

All the examinations were performed at slice collimation 5 mm.

Based on the effective doses received by children in specific age groups calculated in the study, the probability of developing and dying from leukemia or any other cancer was calculated based on BEIR VII [11].

However, to assess the importance of exposure for the health of the studied children, it is necessary to estimate the effective dose, which allows estimating the probability of stochastic effects. The effective dose can be estimated from the DLP value using conversion factors. These coefficients were decided based on the results of simulation calculations, considering the location of the areas (body parts) that were exposed and the size of the patient, which in the case of children is related to their age. In simulation calculations, patients are imitated by mathematical phantoms. This study uses data published in Lee et al. [12] and IARC document [13] relating to head examinations in children of different ages.

In this way, the effective dose can be expressed as:


(1) $$\text{E}=\text{DLP}\times {\text{K}}_{\text{W}}$$

where:

E – effective dose (mSv),

DLP – dose-length product (mGy × cm),

K_w_ – conversion factor appropriates for a given age group (mSv/mGy × cm).

All statistical calculations were performed in Statistica 13.3 software.

The study was conducted in accordance with the Declaration of Helsinki and approved by the Bioethical Commission of the Medical University of Lodz, Łódź, Poland (approval No. RNN/239/23/KE, October 10, 2023).

## RESULTS

In total, data from 141 patients aged <1 year to 17 years who underwent CT in January 2021 – August 2022 were analyzed.

Table 2 presents data on the average number of CT examinations per patient for each age group.

**Table 2.** Average number of computed tomography (CT) examinations per patient for each age group of children and adolescents <18 years, treated for hydrocephalus and subjected to CT at pediatric reference center, Poland, January 2021 – August 2022

Age group	Patients (N = 141) [n]	Tests [n] (M±SD)
<1 year	17	2.3±1.9
1–3 years	37	1.6±0.9
4–10 years	37	1.9±1.7
≥11 years	50	1.9±1.7

Average DLP values were calculated for patients from specific age groups. They are presented in Table 3.

**Table 3.** Average dose-length product (DLP) values in individual age groups of children and adolescents <18 years, treated for hydrocephalus and subjected to CT at pediatric reference center, Poland, January 2021 – August 2022

Age group	Age range [years]	Tests* [n] (M)	DLP [mGy × cm] (M±SD)
<1 year	1	2	342±74
1–3 years	2	2	504±145
4–10 years	7	2	716±114
≥11 years	7	2	1029±134

* Rounded to whole numbers.

The effective dose values estimated in this way for patients from age groups are presented in Table 4. The values given above in Table 4 should be interpreted as follows:

– the child <1 year old receives an average effective dose of 4.2 mSv,
– the child 1–3 years old receives an effective dose of 10.5 mSv, and in each subsequent year of this interval a dose of 3.1 mSv is received,
– the child 4–10 years old receives an effective dose of 16.0 mSv, and in each subsequent year of this interval, a dose of 0.8 mSv is received,
– the patient 11–17 years old receives an effective dose of 20.8 mSv, and in each subsequent year of this interval, a dose of 0.7 mSv is received.

**Table 4.** Estimated effective doses (E) for patients in specific age groups based on mean dose-length product (DLP) values – study among children and adolescents <18 years, treated for hydrocephalus and subjected to computed tomography at pediatric reference center, Poland, January 2021 – August 2022

Age group	Kw [mSv/mGy × cm]	E [mSv] (M±SD)	
		the entire age range	per 1 year of life
<1 year	0.0062	4.2±0.9	4.2±0.9
1–3 years	0.0062	6.3±1.8	3.1±0.9
4–10 years	0.00383*	5.5±0.9	0.8±0.1
11–17 years	0.00233	4.8±0.6	0.7±0.1

Kw – conversion factor.

* Value calculated as the average for the age groups 4–6 years old and 9–11 years old included in the work [13].

The mentioned list of dose values means that the greatest radiation burden is experienced by children <1 year of age in whom hydrocephalus has just been detected and is being diagnosed.

If control (or other therapy) is continued in the subsequent years of the patient's life, each subsequent year adds a specific effective dose, although the frequency of CT examinations decreases significantly, as do the values of effective doses.

As a result, by the age of 17 years, a patient with hydrocephalus diagnosed beginning from infancy may receive a total effective dose of almost 21 mSv.

Tables 2–4 show the mean DLP and effective dose values. However, the source data (which are not included in the paper due to their considerable volume) include a number of cases of patients who had CT scans performed more than twice in the period of 2–11 months:

– in the age group of children <1 year old, there were 3 cases with a number of tests ranging from 5 to 7,
– in the group 1–3 years old there were 6 cases with 3–4 tests,
– in the group 4–10 years old there were 8 cases with the number of tests ranging from 3 to 7,
– in the group ≥11 years old, there were 12 patients with the number from 3 to even 9 tests.

The most CT examinations were performed in a child aged 3–4 years, who had 11 CT examinations over 11 months. Table 5 shows the calculated probability of induction and death from leukemia or other cancer by gender, for the individual age groups analyzed in the study. The number of cases per 100 000 people irradiated with the doses presented in Table 4 was calculated.

**Table 5.** Number of incidents and deaths from leukemia or any other cancer per 100 000 persons for specific age groups following receipt of the doses specified in Table 4 – study among children and adolescents <18 years, treated for hydrocephalus and subjected to CT at pediatric reference center, Poland, January 2021 – August 2022

Age group	Lifetime attributable risk [n/100 000 persons]							
	cancer incidence				cancer morality			
	leukemia		all cancers		leukemia		all cancers	
	males	females	males	females	males	females	males	females
<1 year	14	8	108	201	3	2	46	74
1–3 years	24	20	211	531	9	7	126	202
4–10 years	8	30	94	908	21	15	235	369
11–17 years	5	23	61	642	21	15	184	281

## DISCUSSION

In the analyzed CT studies the exposure covers only the head, in children receiving a significant effective dose is more important than in the case of adult patients, because in children (especially in the early years of life) a significant part of the red bone marrow is located in the bones of the skull.

According to data published in Abu-Gheida et al. [14], in a child aged 2–3 months, the skull bones contain 30% of the total mass of red bone marrow, while by the age of 5 years – this content decreases to 20% (this justifies the differences in the values of conversion coefficients K_w_ for specific age groups). Nevertheless, in a child <4 years of age, approx. 25% of the red bone marrow is located in the repeatedly irradiated skull, which significantly increases the probability of inducing leukemia (Table 5).

The calculated probabilities of leukemia and other cancers show that children in the first 4 years are most at risk of developing leukemia and other cancers, regardless of gender. However, the probability of dying from it remains constant throughout the teenage years. This is consistent with the available literature [15].

In addition to the discussion of the DLP results presented in Table 3, it should be noted that despite the use of standard testing protocols assigned to specific age groups, the coefficient of variation of DLP is relatively high: 13–29%. The explanation is probably the natural variation in the body structure of children in the same age group.

The assessment of hydrocephalus as the cause of numerous CT examinations in children treated at pediatric reference center was carried out 16 years ago, and the results of this assessment were published in Rybka et al. [16]. Computed tomography examinations were then performed using a fourth-generation single-row scanner PICKER-2000 (Picker International, Cleveland, OH, USA). The software of this scanner did not include dedicated pediatric protocols, so children were examined according to the same protocols as adult patients. Data on 380 patients aged from a few months to 19 years, examined for hydrocephalus in 1996–2006, were analyzed. It was found that on average 7 CT examinations were performed for 1 patient – without indicating the age range. The result referred to the entire period of patient observation during the treatment process. The distribution of the number of tests performed showed that 50% of patients were tested 2–7 times, 28% – 7–15 times, and 5% were tested >15 times (during the observation period). The comparison of the results of the currently reported study and the analysis at that time shows that the frequency of CT examinations ordered in the treatment of hydrocephalus in children has clearly decreased, which certainly has a positive impact on the radiation safety of young patients.

## CONCLUSIONS

Based on the results collected as part of this analysis, the following conclusions can be drawn:

– radiation exposure of children undergoing head CT scans in connection with the diagnosis and treatment of hydrocephalus is now minimized thanks to the introduction of pediatric protocols adapted to the age of patients;
– the frequency of ordered CT head examinations has decreased radically in favor of magnetic resonance imaging, which proves the medical staff ‘s awareness of the patient's radiological protection;
– in the process of diagnosing hydrocephalus, the highest effective doses are given to children <1 year of age, which generates a higher probability of induction of leukemia or other cancers.

Although the need to monitor the course of hydrocephalus treatment is obvious, special attention should be paid to the selection of CT examination parameters to ensure a reasonably possible reduction of the dose received by the young patient.
